# Supplementary material for: Understanding the Metabolism and Dissipation Kinetics of Flutriafol in Vegetables under Laboratory and Greenhouse Scenarios
Source: Foods. 2023 Jan 2;12(1):201. doi: 10.3390/foods12010201 (PMC9818287; doi:10.3390/foods12010201)
Supplement: Supplementary file 1 [file foods-12-00201-s001.zip › foods-2055372-supplementary.pdf]

SUPPLEMENTARY MATERIAL to the article: Understanding the metabolism and dissipation kinetics of flutriafol in vegetables under laboratory and greenhouse scenarios

María Elena Hergueta-Castillo, Rosalía López-Ruiz, Antonia Garrido Frenich and Roberto Romero-González*

Department of Chemistry and Physics (Analytical Chemistry Area), Research Centre for Mediterranean Intensive Agrosystems and Agri-Food Biotechnology (CIAIMBITAL), Agrifood Campus of International Excellence ceiA3, University of Almería, E-04120 Almería, Spain

ORCID Codes:
María Elena Hergueta-Castillo: 0000-0002-4539-8855
Rosalía López-Ruiz: 0000-0003-0806-9013
Antonia Garrido Frenich: 0000-0002-7904-7842
Roberto Romero-González: 0000-0002-2505-2056

* Corresponding author: rromero@ual.es

Table of contents

Table S1. Parameters of greenhouse experiment.

Table S2. Concentration (µg/kg) of unknown flutriafol metabolites under laboratory conditions at single dose.

Table S3. Concentration (µg/kg) of unknown flutriafol metabolites under laboratory conditions at double dose.

Figure S1. Extracted Ion Chromatograms of triazol alanine at 48 h under laboratory conditions: a) single dose; b) double dose; c) full Scan MS experimental spectrum of single dose; d) full Scan MS experimental spectrum of double dose; and e) full Scan MS theoretical spectrum.

Table S1. Parameters of greenhouse experiment †

**Study location**	Retamar, Almeria, Spain
**Orientation**	East-West
**Area**	727.2 m^2
**Working field**	156 plants
**Agricultural model**	Passive climate control systems with 4% ventilation and side windows (12.9% ventilation)
**Cultivation system**	Hydroponic crop
**Irrigation water**	0.6-3.0 dS/m
**Application dose**	0.090 L/h
**Air temperature (greenhouse indoor)**	16.5ºC
**Binomial plant name**	Solanum lycopersicum L.(tomato)
**Plant stage at pesticide application**	Bloom
**BBCH scale**	60

† Abbreviations: BBCH-scale: Biologische Bundesanstalt, Bundessortenamt und CHemische Industrie, used to identify the phenological development stages of plants.

Table S2. Concentration (µg/kg) of unknown flutriafol metabolites under laboratory conditions at single dose †

Metabolite	Sampling period (days)										
	**2 h**	**6 h**	**1**	**2**	**5**	**10**	**15**	**22**	**29**	**36**	**43**
MF1	--	--	--	--	--	--	--	--	2.7	--	2.9
MF3	--	--	--	--	$<\text{LOQ}_f$	--	--	$<\text{LOQ}_f$	--	--	--
MF4	--	--	--	--	$<\text{LOQ}_f$	--	$<\text{LOQ}_f$	--	$<\text{LOQ}_f$	$<\text{LOQ}_f$	--

† Abbreviations: MF1: metabolite 2; MF3: metabolite 3; MF4: metabolite 4; $<\text{LOQ}_f$: compound detected below limit of quantification of flutriafol (2 µg/kg), but not quantified; --: compound not detected.

Table S3. Concentration (µg/kg) of unknown flutriafol metabolites under laboratory conditions at double dose †

**Metabolite**	**Sampling period (days)**										
	**2 h**	**6 h**	**1**	**2**	**5**	**10**	**15**	**22**	**29**	**36**	**43**
MF1	--	--	--	--	--	--	--	2.8	3.1	--	5.6
MF3	--	--	--	--	$<LOQ_f$	--	--	$<LOQ_f$	--	--	--
MF4	--	--	--	--	$<LOQ_f$	$<LOQ_f$	$<LOQ_f$	$<LOQ_f$	$<LOQ_f$	$<LOQ_f$	$<LOQ_f$

† Abbreviations: MF1: metabolite 2; MF3: metabolite 3; MF4: metabolite 4; $<LOQ_f$: compound detected below limit of quantification of flutriafol (2 µg/kg), but not quantified; --: compound not detected.

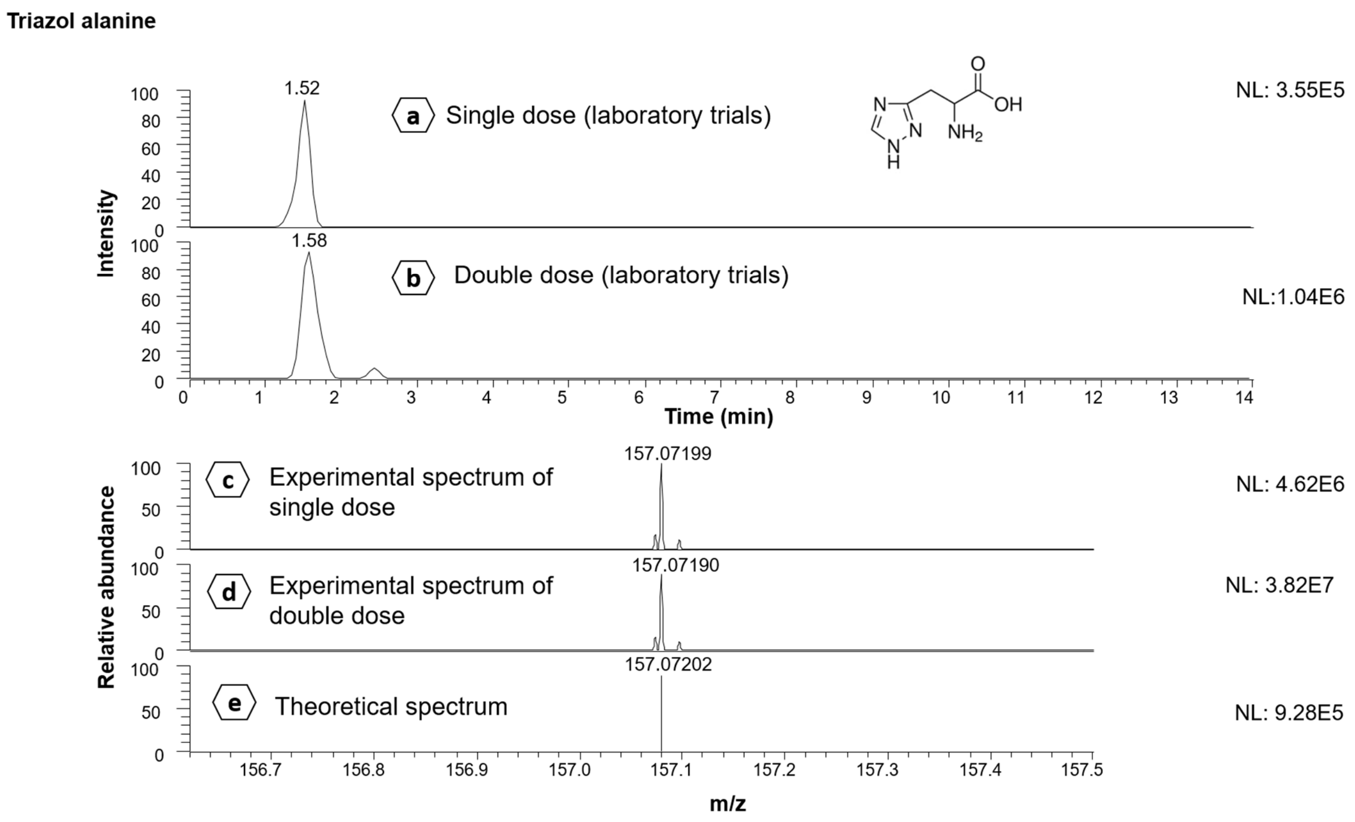

Figure S1. Extracted Ion Chromatograms of triazol alanine at 48 h under laboratory conditions: a) single dose; b) double dose; c) full Scan MS experimental spectrum of single dose; d) full Scan MS experimental spectrum of double dose; and e) full Scan MS theoretical spectrum
